# Supplementary material for: Anti-SOD1 Nanobodies That Stabilize Misfolded SOD1 Proteins Also Promote Neurite Outgrowth in Mutant SOD1 Human Neurons
Source: Int J Mol Sci. 2022 Dec 16;23(24):16013. doi: 10.3390/ijms232416013 (PMC9784173; doi:10.3390/ijms232416013)
Supplement: Supplementary file 1 [file ijms-23-16013-s001.zip › ijms-2048107-supplementary.pdf]

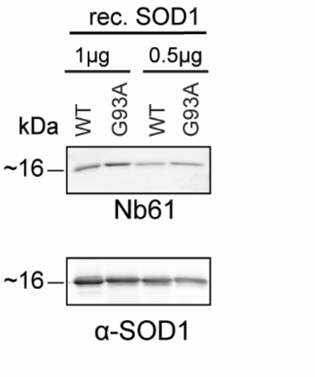

Figure S1. Nb61 reacts with denatured SOD1 WT and G93A. Recombinant (rec.) SOD1 WT and G93A at 1 μg or 0.5 μg per lane were subjected to denaturing SDS-PAGE Western blot analysis and probed with Nb61 (top). As a positive control, a duplicate immunoblot was probed with a commercial pan anti-SOD1 antibody (bottom). The Western blots were processed as described in the methods.

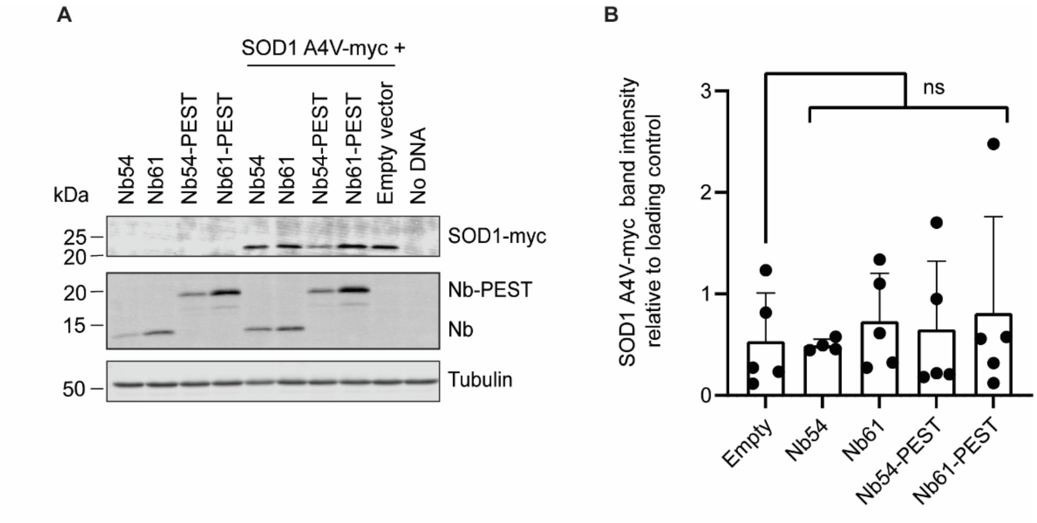

Figure S2. Western analysis of SOD1-myc levels upon co-transfection with nanobody constructs**. (A)** HEK293T cells were transfected with the indicated nanobody constructs or empty vector control and SOD1 A4V-myc and subjected to Western blot analysis. The blots were probed with anti-myc, anti-nanobody and anti-tubulin (as a loading control) antibodies. **(B)** Quantification of SOD1-myc band intensity in samples co-transfected with SOD1 A4V-myc and nanobody constructs. Each point in the graph represents an individual experiment. Error bars depict standard deviation. Kruskal-Wallis with Dunn's multiple comparison test was used to compare samples co-transfected with SOD1 A4V-myc and empty vector control.
